# An evaluation of the Kodak Ektachem system for the determination of glucose and urea

**DOI:** 10.1155/S1463924679000766

**Published:** 1979

**Authors:** R. Haeckel, O. Sonntag, K. Petry

**Affiliations:** Institut fur Chemie Medizinische Hochschule Hannover Germany


them and then using these results to set the corresponding monitor readings at the correct level. Once locked-in in this way changes would only be needed if the stack conditions were altered or some significant change made in the sampling procedure.

### Conclusion

The design and evaluation of an automated instrument for the continuous monitoring of lead emissions from stacks is described. This is one of a number of such monitors being designed and evaluated for the Alkali Inspectorate in their search for a reliable, economical instrument for the continuous monitoring of emissions of metal dust and fume from stacks and ducts. The basic concept of the described instrument is seen to be valid but certain problems have been discovered during the evaluation stage. Until the modifications discussed above have been made, and the monitor retested on-site, it is not possible to say that a proven instrument, based on the concepts described, is currently available.

### An evaluation of the Kodak Ektachem system for the determination of glucose and urea *

R. Haeckel and O. Sonntag
Technical Assistant: K. Petry

Institut fur Chemie, Medizinische Hochschule, Hannover, West Germany.

### Introduction

The Kodak Ektachem analytical system is a unique concept recently introduced into the field of clinical chemistry, which applies the company's considerable experience in photography. In contrast to conventional clinical analysis, the chemistry is carried out on a thin film of interacting chemical layers termed a slide. In the analytical instrument produced by Kodak, a dispenser places a drop of serum onto the slide which is then moved into an incubator to develop the colour reaction. The measurements are carried out by reflectance densitometry.

### Thin film chemistry

The principles of this technique can be described by reference to the analysis of urea. Figure 1 shows a schematic diagram of the slide used and also illustrates the chemistry integrated into the multilayers. The spreading layer is an isotropically porous non-fibrous layer with an 80% void volume and a mean pore size of 1.5 microns. The spreading and metering action which is aided by surfactants, compensates for any differences in sample size (nominally 10µl are applied) and serum viscosity. A constant volume per unit area is then naturally applied to subsequent layers of the slide. High molecular weight materials, such as protein, are removed by this layer and consequently do not interfere with the subsequent analysis. Titanium dioxide is incorporated in the spreading layer to improve its reflectivity.

It also acts as a white background for reflectance measurements against which the colour density produced in the indicator layer can be measured. The reagent layer contains the urease which catalyzes the hydrolysis of urea in the sample to produce ammonia. Water from the serum sample applied, swells the gel allowing the urea to diffuse into the layer. The layer is buffered to pH 7.8 and this maintains the ammonia at a low level, and subsequently extends the range of the assay. A third layer consisting of cellulose acetate butyrate with selective permeability, allows non-ionic materials such as ammonia and water to pass through to the indicator layer. Ionic compounds are excluded, providing some degree of selectivity. The indicator layer consists of a gel-binder incorporating the indicator reagent, in this example, N - propyl - 4 - ( 2, 6 - dinitro- 4 - chlorobenzyl ) - quinolinium ethane sulphonate. Free ammonia which diffuses into this layer reacts with the indicator to form a dye which has a molar absorptivity of approximately 5000 and a broad absorption peak at 520 nm. The reflectance density is measured off the peak at 670 nm. The final layer is a clear polyester support upon which all the other layers are coated. It is transparent and allows measurement of the dye density formed in the indicator layer. Slides for urea and glucose determination are also available. Research has been strongly stimulated by the knowledge that Kodak had developed the multilayer film technique and many more methods are forthcoming.

The authors' experiences with the technique for the determination of the concentration of glucose and urea in serum are presented in this paper. The experimental work was carried out using an investigational unit over a four month period. The evaluation was directed towards assessing the analytical reliability of the thin film concept rather than to the technical reliability and practicality of the instrument used.

In the technique described in detail elsewhere [1, 2], drops of serum are directly applied to the analytical slides. Relatively high concentrations of the analytes to be determined, and also any interferences in the undiluted sample, are therefore in contact with the reagents held in the individual layers. In conventional techniques, samples are usually diluted by factors of 1:10 or 1:1000. Special attention in this evaluation was focussed on determining any possible interferences due to various exogenic and endogenic compounds present in the serum samples.

* Translated from the German by R. Arndt and P.B. Stockwell. The German text of this paper will be published in part in the November 1979 issue of G-I-T Labormedizin.

Since reactions in this technique take place in the thin films physical parameters such as difusion, capillary adhesion and viscosity attain a new importance in clinical chemistry. Unlike absorbance, end measurement by reflectance is not a linear function of concentration.

### Methodology

### Glucose slide

The method for determination of glucose concentration is similar to the method described by Trinder [3]. $H_2O_2$ is formed in the presence of glucoseoxidase (EC 1.1.3.4.) and is determined by a subsequent indicator reaction with peroxidase (EC 1.11.1.7.). However, phenol is replaced by 1,7-dihydroxynapthalene (DHN). Details on the various layers in the film are shown in Table 1.

The wavelength maximum of colour absorption appears at a wave length of 495 nm, but in order to improve the linearity of the measurement to reduce interference by bilirubin, slides are measured at 540 nm in this system. The slide reaction takes place rapidly, however there is also a slow mutarotation of $\alpha$ to $\beta$–glucose. After 7 minutes 80% of the total glucose is converted to product.

### Urea slide

The method for determination of urea concentration, has already been described above. The five layers of the film are summarized in Table 2. In the urea also the reaction does not proceed to completion.

The Ektachem system was operated according to the instructions given by the manufacturer. The following comparative methods were used. A kinetic assay of urea using the urease-GLDH-principle on a Gemsaec analyser [4], a glucose assay using the hexokinase method after Somogyi deproteinization on an endpoint automatic analyzer 5030 from Eppendorf Gerätebau GmbH [5]. In addition, reference was made to an assay using the hexokinases method on a SMA 12/60 of Technicon GmbH (D-6368 Bad Vilbel).

**Table 1 Glucose method**

| | | |
|---|---|---|
| spreading layer | cellulose-acetate pigmented with $TiO_2$ | |
| | thickness | 100 $\mu$m |
| | average diameter of pores | 1,5 $\mu$m |
| reagent layer | gelatine | |
| | thickness | 10mm |
| | pH | 4,8 |
| | GOD (Aspergillus niger) | |
| | POD (horse-radish) | |
| | 4- aminophenazon (4-aminoantipyrin) | |
| | 1,7-DHN | |
| substrate | polyethylene-terephtalate | |
| stability | at least 1 year at +4°C | |

**Table 2 The 5 thin film layers of the urea slide**

| | | | |
|---|---|---|---|
| 1 | spreading layer | $TiO_2$-cellulose-acetate | |
| 2 | reagent layer | urease | |
| | | gelatine | |
| | | pH = 7.8 | |
| 3 | semipermeable layer | cellulose-acetate-butyrate | (0.5 $\mu$m) |
| 4 | indicator layer | Merocyanin1) in cellulose-acetate | |
| 5 | substrate | polyethylene-terephtalate | |

1) *N-propyl-4-(2,6-dinitro-4-chlorobenzyl) quinolinethane-sulfonate (pH-indicator)*

### Interferences

Interferences have been tested in a similar manner to that reported earlier [6]. A reference pool of serum was divided into several samples to which various pharmaceuticals (pure compounds) were added. A control sample (i.e. no compounds added) was also prepared from the batch. Pharmaceuticals were applied in concentrations ten times lower than those used in earlier studies [6, 7] in order to correspond more closely to actual therapeutic conditions. From the results obtained using the control samples, the range of tolerance was determined (mean value ±3 standard deviations). Any interference due to proteins in human serum was tested using samples with pathological composition of

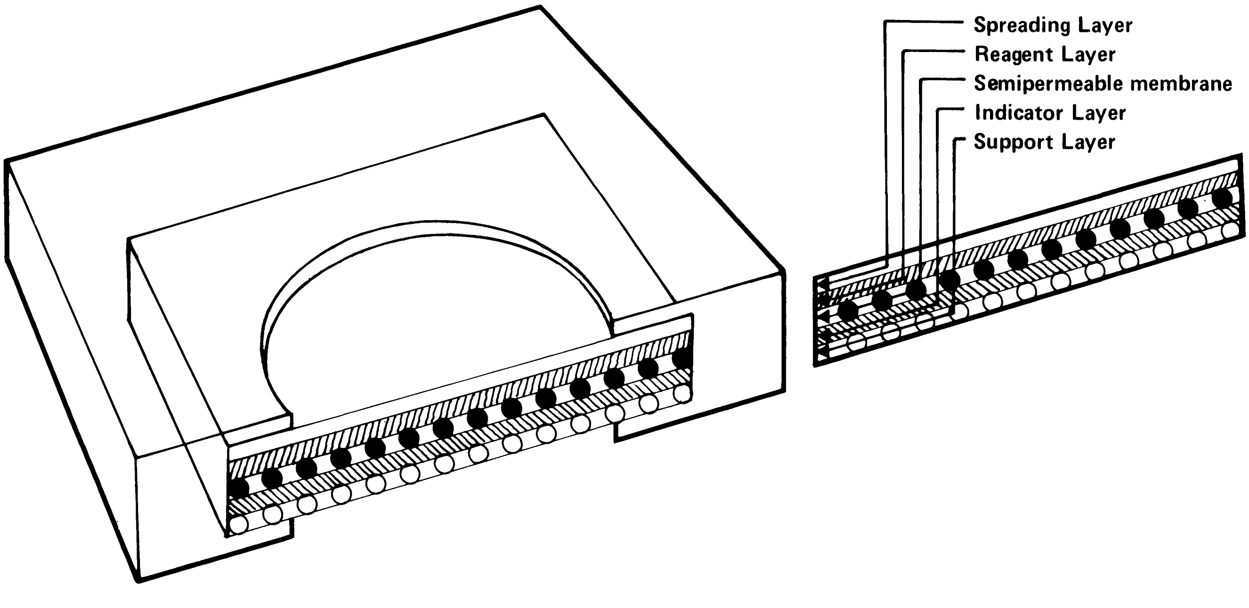

*Figure 1. Schematic diagram of the slide used, illustrating the chemistry integrated into the multilayers.*

proteins, and with solutions of albumin (Rinder-Albumin reinst, Behringswerke AG, D-3550 Marburg).

Interferences due to endogenic chromogenes (bilirubin, haemoglobin, turbidity) were investigated using comparative measurements, outlined above [4, 5]. The corresponding analytes were added to human sera in pathological concentrations.

### Statistics

The precision was determined using the scheme described in the NCCLS report [8]. Three control sera with different concentrations (Tables 3 and 4) were determined using a fixed permutational scheme [8]. They were determined in two daily series throughout a 20 day period, and results were performed in duplicate.

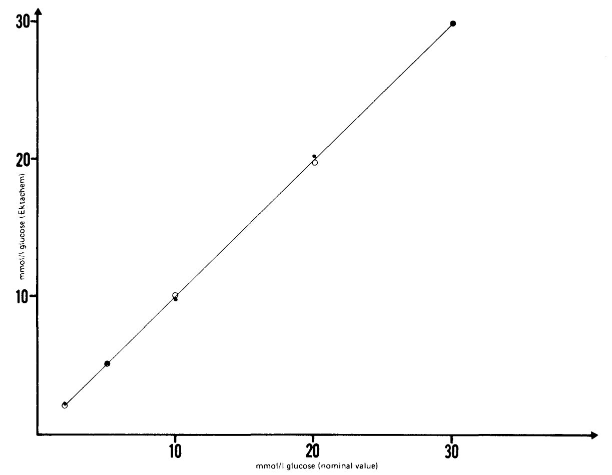

*Figure 2. Linear range for determination of glucose with aqueous (○–○) and albuminic solutions of glucose (●–●). The calibration was carried out with 3 glucose solutions (3, 15, 30 mmol/l) with resp. without albumin.*

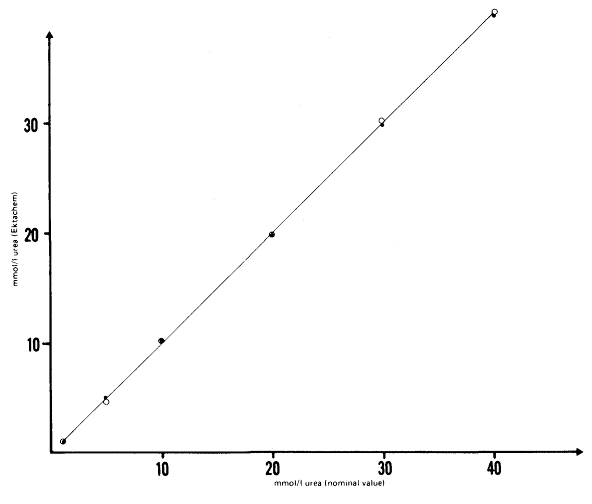

*Figure 3. Linear range for determination of urea with aqueous (○–○) and albuminic solutions of urea (●–●). The calibration was carried out with 3 urea solutions (5, 20, 40 mmol/l) with resp. without albumin.*

**Table 3 Day-to-day precision of determination of glucose concentrations with the Ektachem system using the NCCLS recommendations.**

| serum | run 1=morning 2=afternoon | position in run | mean value | S | coeff var | n |
|---|---|---|---|---|---|---|
| 1 High control' | 1 | 1 | 28,38 | 0,45 | 1,60 | 20 |
| 2 Geo A | | 2 | 26,08 | 0,49 | 1,88 | 19 |
| 3 Geo A | | 3 | 26,07 | 0,49 | 1,88 | 21 |
| 4 High control' | 2 | 1 | 28,52 | 0,44 | 1,53 | 20 |
| 5 Geo A | | 2 | 26,23 | 0,58 | 2,20 | 22 |
| 6 Geo A | | 3 | 26,19 | 0,50 | 1,92 | 22 |
| 7 Low control' | 1 | 1 | 3,38 | 0,11 | 3,25 | 21 |
| 8 Pathonorm L | | 2 | 1,43 | 0,10 | 6,99 | 21 |
| 9 Pathonorm L | | 3 | 1,42 | 0,11 | 7,75 | 21 |
| 10 Low control' | 2 | 1 | 3,37 | 0,10 | 2,97 | 21 |
| 11 Pathonorm L | | 2 | 1,42 | 0,11 | 7,75 | 21 |
| 12 Pathonorm L | | 3 | 1,42 | 0,10 | 7,04 | 21 |
| 13 Wellcomtrol II' | 1 | 1 | 11,65 | 0,20 | 1,72 | 20 |
| 14 Wellcomtrol I'' | | 2 | 6,29 | 0,13 | 2,07 | 20 |
| 15 Wellcomtrol I | | 3 | 6,29 | 0,13 | 2,07 | 20 |
| 16 Wellcomtrol II' | 2 | 1 | 11,63 | 0,23 | 1,98 | 20 |
| 17 Wellcomtrol I'' | | 2 | 6,29 | 0,12 | 1,91 | 20 |
| 18 Wellcomtrol I | | 3 | 6,30 | 0,09 | 1,43 | 19 |
| 19 Pathonorm L'' | 1 | | 1,42 | 0,10 | 7,04 | 20 |
| 20 Pathonorm L'' | 2 | | 1,41 | 0,11 | 7,80 | 21 |
| 21 High control''' | 1 | | 28,33 | 0,47 | 1,66 | 11 |
| 22 High control''' | 2 | | 28,46 | 0,50 | 1,76 | 14 |

*' including a possible cross contamination (concentration of preceding sample differed)*
*'' including a possible cross contamination due to change from high to low concentration*
*''' including a possible cross contamination due to change from low to high concentration*

**Table 4 Day-to-day precision of determination of urea concentrations with the Ektachem system using the NCCLS recommendations**

| serum | run 1= morning 2=afternoon | position in run | mean value | S | coeff var | n |
|---|---|---|---|---|---|---|
| 1 High control* | 1 | 1 | 37,52 | 0,70 | 1,86 | 20 |
| 2 Geo A | | 2 | 31,98 | 0,46 | 1,43 | 19 |
| 3 Geo A | | 3 | 31,75 | 0,47 | 1,49 | 21 |
| 4 High control* | 2 | 1 | 37,74 | 0,74 | 1,96 | 21 |
| 5 Geo A | | 2 | 31,91 | 0,66 | 2,06 | 22 |
| 6 Geo A | | 3 | 31,99 | 0,50 | 1,56 | 22 |
| 7 Low control* | 1 | 1 | 3,26 | 0,11 | 3,43 | 20 |
| 8 Pathonorm L | | 2 | 2,94 | 0,12 | 3,91 | 22 |
| 9 Pathonorm L | | 3 | 2,92 | 0,10 | 3,51 | 22 |
| 10 Low control* | 2 | 1 | 3,27 | 0,11 | 3,44 | 21 |
| 11 Pathonorm L | | 2 | 2,93 | 0,11 | 3,78 | 22 |
| 12 Pathonorm L | | 3 | 2,93 | 0,11 | 3,72 | 22 |
| 13 Wellcomtrol II* | 1 | 1 | 29,44 | 0;49 | 1,67 | 21 |
| 14 Wellcomtrol I** | | 2 | 9,12 | 0,23 | 2,50 | 22 |
| 15 Wellcomtrol I | | 3 | 9;07 | 0,24 | 2,64 | 22 |
| 16 Wellcomtrol II* | 2 | 1 | 29,44 | 0,46 | 1,55 | 21 |
| 17 Wellcomtrol I** | | 2 | 9,09 | 0,25 | 2,71 | 22 |
| 18 Wellcomtrol I | | 3 | 9,12 | 0,22 | 2,45 | 21 |
| 19 Pathonorm L** | 1 | | 2,94 | 0,10 | 3,28 | 20 |
| 20 Pathonorm L** | 2 | | 2,93 | 0,09 | 3,22 | 22 |
| 21 High control*** | 1 | | 37,47 | 0,61 | 1,62 | 12 |
| 22 High control*** | 2 | | 37,70 | 0,40 | 1,07 | 13 |

** including a possible cross contamination (concentration of preceding sample differed)*
*** including a possible cross contamination due to change from high to low concentration*
**** including a possible cross contamination due to change from low to high concentration*

Within-run imprecision was calculated from the second and third value (position in run, see Table 1), and between-day imprecision from the second, respectively the third value of a triple determination.

Correlation coefficients were calculated using standardized principle component analysis [9, 10]. The samples of serum were selected from those specimens received daily in the authors' central laboratory, approximately 25% of the results were below, 50% within, and 25% above the normal range.

### Results

### Precision

The between-day imprecision with 10 different control sera varied from 0.9% to 2.7% for glucose concentration, and from 1.9% to 3.1% for urea concentration (Table 5).

The concentration of the preceding sample does not have an influence on the precision since as expected, the system works without cross contamination (Tables 3 and 4).

The within-run imprecision is smaller by approximately a factor of 0.3 to 0.7 than the between-day imprecision (Table 6).

### Accuracy

The range of linearity for glucose (1.1 to 33.3 mmol/l) and for urea (0.7 to 42.7 mmol/l) as specified by the manufacturer, was confirmed using aqueous and albumin standard solutions (Figures 2 and 3).

Comparing the various methods used, a very good correlation was obtained with patient sera over the entire range of measurements (Figures 4 and 7). The mean values between Ektachem and the comparative method ($\bar{x}$ and $\bar{y}$ in

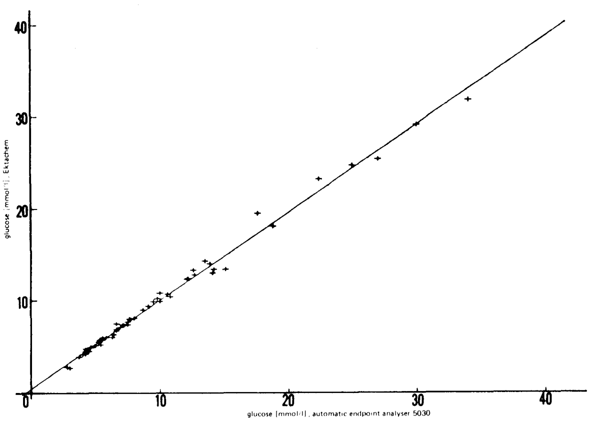

*Figure 4. Comparative determination of glucose in sera of patients using the Ektachem system and the Eppendorf endpoint analyzer 5030. Analysis of principle component: $\bar{y}=0.96\ x + 0.46$; $\bar{x}= 8.72$; $\bar{y}= 8.79$; $r = 1.00$; $n = 74$; t-test for paired values: $t= -1.08$; $p > 0.05$.*

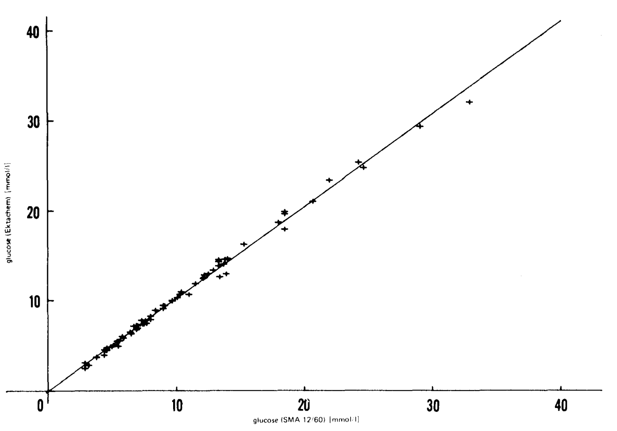

*Figure 5. Comparative determination of glucose in sera of patients using the Ektachem system and the SMA 12/60. Analysis of principle component: $y = 1.02\ x - 0.05$; $\bar{x}= 7.51$; $s = 4.74$; $\bar{y}= 7.64$, $s = 4.87$; $r = 1.00$; $n = 171$. t-test for paired values: $t = -4.84$; $p > 0.05$.*

**Table 5 Day-to-day precision of glucose and urea determination with the Ektachem system (VK = coefficient of variation). In 10 days the concentrations (mmol/l) in various control sera were determined in duplicate analyses (a,b).**

| control serum (batch number) | pH-value after at most 2h after reconstituation | glucose | | | urea | | |
|---|---|---|---|---|---|---|---|
| | | given value | mean value1) | VK1) | given value | mean value1) | VK1) |
| Precilip (661) | 8,57 | 6,83 | a 6,54 | 1,30 | 6,84 | 6,00 | 2,16 |
| | | | b 6,54 | 0,94 | | 6,00 | 1,96 |
| Seronorm (135) | 7,71 | 6,70 | a 6,55 | 2,13 | 13,4 | 11,72 | 2,75 |
| | | | b 6,55 | 1,40 | | 11,87 | 2,17 |
| Validate N (0527087) | 7,55 | 4,40 | a 4,40 | 1,70 | 6,5 | 5,72 | 2,81 |
| | | | b 4,45 | 1,65 | | 5,76 | 2,63 |
| Kontrollogen L (3105 A) | 7,37 | 5,33 | a 4,62 | 2,04 | 5,8 | 5,48 | 2,83 |
| | | | b 4,64 | 1,69 | | 5,50 | 2,88 |
| Normosic (414 D) | 7,35 | 5,39 | a 5,15 | 1,15 | 6,24 | 4,85 | 2,91 |
| | | | b 5,16 | 1,93 | | 4,81 | 2,93 |
| Hyland P (P 11) | 6,81 | 11,40 | a 10,89 | 1,93 | 15,0 | 12,54 | 1,59 |
| | | | b 10,98 | 1,76 | | 12,50 | 2,56 |
| Hyland N (NO 4) | 7,75 | 4 25 | a 4,09 | 1,75 | 4,7 | 4,19 | 3,06 |
| | | | b 4,10 | 1,82 | | 4,18 | 3,09 |
| Pathotrol E (PT 75 G) | 5,63 | 13,20 | a 13,26 | 1,69 | 10,2 | 7,81 | 2,09 |
| | | | b 13,31 | 1,27 | | 7,86 | 2,01 |
| Monitrol II (PTD 58 A) | 7,07 | 11,6 | a 11,72 | 2,19 | 11,7 | 10,32 | 2,83 |
| | | | b 11,76 | 2,02 | | 10,28 | 2,27 |
| Monitrol I (147 B) | 7,96 | 4,88 | a 5,11 | 2,67 | 4,83 | 4,43 | 2,92 |
| | | | b 5,15 | 1,96 | | 4,42 | 3,14 |

**Table 6 Precision in the series of glucose and urea determinations with the Ektachem system (VK - coefficient of variation)**

| method | control serum | batch | given value (mmol/l) | x̄ (mmol/l) | V.K. % | n |
|---|---|---|---|---|---|---|
| glucose | Pathonorm L | 15 | 1,39 | 1,40 | 0,79 | 15 |
| | High Control | | 28,3 | 28,60 | 0,41 | 15 |
| | Validate N | 0845058 | 4,4 | 4,43 | 0,98 | 15 |
| | Monitrol II | PTD 58 A | 11,6 | 11,74 | 9,63 | 15 |
| urea | Pathonorm L | 15 | 3,50 | 2,82 | 1,45 | 15 |
| | High Control | | 37,3 | 36,82 | 0,58 | 15 |
| | Validate N | 0845058 | 6,5 | 5,75 | 1,17 | 15 |
| | Monitrol II | PTD 58 A | 11,7 | 10,30 | 1,74 | 15 |

**Table 7 Comparative determination of concentrations of glucose and urea in sera of patients with lowered creatinine clearance**

| patient | glucose (mmol/l) | | urea (mmol/l) | | creatinine clearance ml – $min^{-1}$ |
|---|---|---|---|---|---|
| 13 | 6,9 | 6,5 | 42,6 | 42,3 | 1 |
| 282 | 3,4 | 3,5 | 18,5 | 18,1 | 9 |
| 284 | 4,8 | 4,7 | 18,7 | 18,9 | 17 |
| 375 | 4,6 | 4,4 | 13,5 | 13,4 | 20 |
| 376 | 4,5 | 4,2 | 32,0 | 32,7 | 4 |
| 523 | 4,6 | 4,3 | 22,0 | 21,3 | 9 |
| 143 | 5,3 | 5,4 | 32,1 | 32,7 | 10 |
| 562 | 4,4 | 4,3 | 21,8 | 21,9 | 14 |
| 556 | 5,4 | 5,3 | 20,4 | 20,3 | 12 |
| 163 | 3,1 | 2,9 | 2,1 | 2,2 | 8 |
| 162 | 5,5 | 5,5 | 13,2 | 13,3 | 7 |
| 175 | 7,1 | 7,2 | 3,2 | 3,3 | 10 |
| 179 | 5,8 | 5,7 | 12,5 | 12,8 | 13 |
| 253 | 3,9 | 3,9 | 17,3 | 17,2 | 7 |
| 351 | 4,7 | 4,6 | 20,5 | 20,1 | 9 |
| 420 | 2,5 | 2,4 | 12,7 | 12,9 | 3 |
| 423 | 7,8 | 7,9 | 13,8 | 14,0 | 4 |
| 102 | 4,9 | 5,0 | 15,2 | 15,5 | 15 |
| 110 | 5,3 | 5,5 | 32,5 | 32,2 | 10 |
| 305 | 6,7 | 6,4 | 19,3 | 19,3 | 2 |
| mean value | 5,06 | 4,98 | 19,20 | 19,22 | |

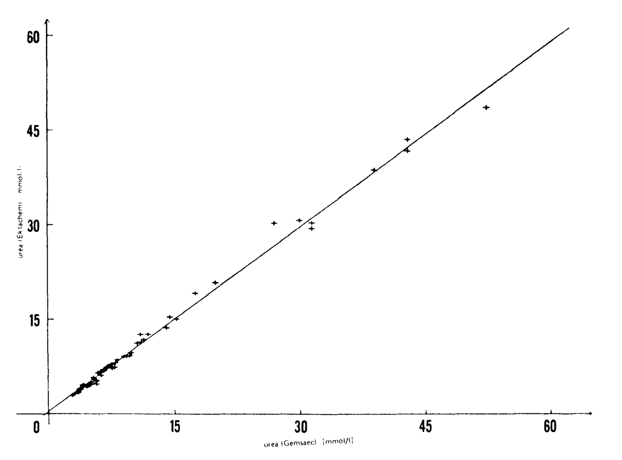

*Figure 6. Comparative determination of urea in sera of patients using the Ektachem system and Gemsaec. Analysis of principle component: $y = 0.99\ x + 0.13$; $\bar{x} = 9.66$; $\bar{y} = 9.73$; $r = 1.00$; $n = 84$. t-test for paired values: $t = -0.80$; $p > 0.05$.*

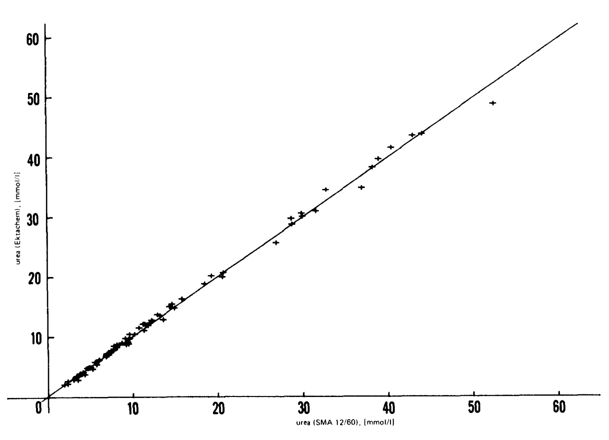

*Figure 7. Comparative determination of urea in sera of patients using the Ektachem system and the SMA 12/60. Analysis of principle component: $y = 0.99\ x + 0.2$; $\bar{x} = 9.02$, $s = 8.97$; $\bar{y} = 9.13$, $s = 8.89$; $r = 1.00$; $n = 171$. t-test for paired values: $t = -2.85$; $p > 0.05$.*

the legends to Figures 4 and 7) differ by less than 1%. This is also true for various samples from patients suffering from kidney deficiency (clearance of creatinine under 20 ml/min), and with high transaminase activities (Tables 7 and 8). Using the Ektachem system a value below the lower limit of determination (1.1 mmol/l) was found in only one serum. This had a glucose value of 1.4 mmol/l. However, this discrepancy could not be resolved.

In tests with 10 different control sera (Table 5), the mean values between Ektachem and the comparative method for the determination of glucose differed by 2%, and for the determination of urea the difference was 14%. The Ektachem value for Pathonorm E, however, was below the value obtained using the urease-GLDH method. A pH value of 5.6 has been measured for this particular control serum (Table 5). It is possible that the buffer capacity in the urea slides was insufficient to compensate for this comparatively low pH value. To overcome this the manufacturer recommends that

**Table 8 Comparative determinations of glucose and urea concentration in sera of patients with increased transaminase activities**

| patient | glucose (mmol/l) | | urea (mmol/l) | | GOT u/l | GPT u/l |
|---|---|---|---|---|---|---|
| 133 | 6,7 | 6,4 | 5,7 | 5,8 | 58 | 129 |
| 210 | 4,9 | 4,7 | 5,3 | 5,2 | 175 | 135 |
| 230 | 4,8 | 4,6 | 4,1 | 3,9 | 664 | 1326 |
| 505 | 4,3 | 4,0 | 3,8 | 3,8 | 64 | 40 |
| 531 | 4,3 | 4,0 | 3,9 | 3,9 | – | 462 |
| 539 | 5,4 | 5,2 | 4,5 | 4,6 | 65 | 50 |
| 088 | 11,6 | 11,7 | 7,2 | 7,1 | 87 | 70 |
| 97 | 5,8 | 5,9 | 7,3 | 7,2 | 44 | 110 |
| 169 | 5,6 | 5,2 | 4,6 | 4,8 | 48 | 52 |
| 130 | 5,3 | 5,5 | 7,1 | 7,2 | 52 | 132 |
| 145 | 6,7 | 6,9 | 8,5 | 8,2 | 70 | 93 |
| 152 | 7,5 | 7,2 | 3,7 | 3,5 | 40 | 120 |
| 205 | 8,1 | 7,8 | 4,5 | 4,2 | 62 | 55 |
| 371 | 4,3 | 4,4 | 3,6 | 3,4 | – | 520 |
| 188 | 2,3 | 2,2 | 3,0 | 3,1 | 62 | 108 |
| 242 | 4,7 | 4,8 | 6,6 | 6,5 | 65 | 75 |
| 363 | 5,4 | 5,6 | 9,3 | 9,2 | 86 | – |
| 361 | 5,3 | 5,2 | 4,3 | 4,2 | 101 | 112 |
| 482 | 5,1 | 5,3 | 1,2 | 1,3 | 112 | 23 |
| 450 | 5,8 | 5,6 | 8,8 | 8,7 | 50 | 90 |
| 047 | 11,8 | 12,0 | 13,6 | 13,7 | 55 | 26 |
| mean value | 5,99 | 5,91 | 5,74 | 5,69 | | |

**Table 9 Refound values of glucose and urea in pool sera to which various components were added. In absence of exogenic compounds mean values ($\bar{x}$) and standard deviations (s) were calculated from 15 determinations. Results of analysis which are outside the range of confidence ($\bar{x} \pm 3s$), are marked with an asterisk. The range of confidence is for glucose 11.14 – 14.08 mmol/l and for urea 6,23 – 6.68 mmol/l.**

| trade name | I.N.N. [1)] | concentration mg/l | glucose (mmol/l) | urea (mmol/l) |
|---|---|---|---|---|
| Amuno | indometacinum | 4 | 13,28 | 6,47 |
| Butazolidin | phenylbutazonum | 12 | 13,06 | 6,51 |
| Metalcaptase | D-penicillaminum | 36 | 12,35 | 6,53 |
| Prolixan | azopropazon-dihydrat | 36 | 13,45 | 6,57 |
| Resochin | chloroquinum | 5 | 12,70 | 6,57 |
| Tanderil | oxyphenbutazonum | 12 | 12,67 | 6,46 |
| Aponal | doxepinum | 6 | 12,76 | 6,43 |
| Megaphen | phenothiazinum | 20 | 13,11 | 6,55 |
| Multum | chlordiazeposidum | 1,8 | 12,26 | 6,56 |
| Aspirin | acidum acetylosalicylicum | 100 | 13,59 | 6,45 |
| Dolviran | acidum acetylocalicylicum, etc. | – | – | – |
| Novalgin | novaminsulfonum | 80 | 12.79 | 6.45 |

1) International designation or recommended by

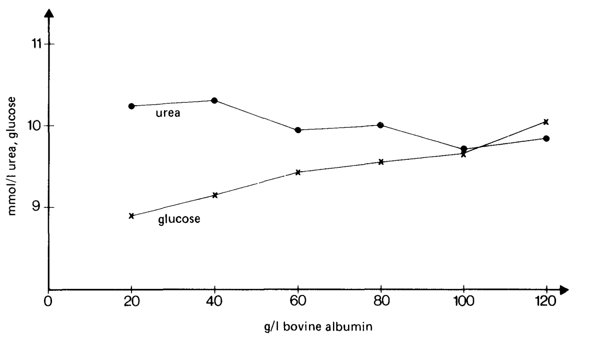

*Figure 8. Influence of albumin on the determination of glucose and urea with the Ektachem system. The glucose and urea concentrations were the same in all aqueous solutions.*

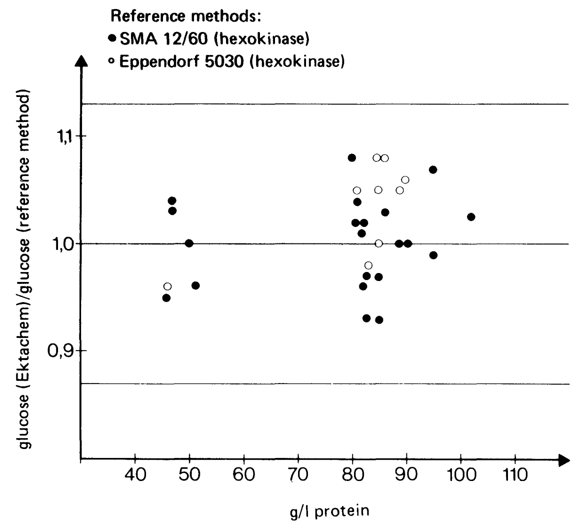

*Figure 9. Influence of protein on determination of glucose concentration with the Ektachem system. The y-axis gives the values of the ratio between glucose (Ektachem value in mmol/l) and glucose (Eppendorf endpoint analyzer 5030 value in mmol/l). The range of confidence corresponds to the ($\bar{x}\pm 3s$) – range ($\bar{x}$ = mean value, s = standard deviation) of the 74 values that were used in Figure 3 and which had been obtained from visually transparent sera.*

the control sera for urea determination is reconstituted with a 20 mmol/l solution of bicarbonate. The experiments presented in Table 3 were performed with lyophilised control sera that had been reconstituted with bidistilled water. Some of the control sera for glucose determination are suitable for use as control measurements (Table 5).

No cross contamination could be detected using the NCCLS procedure (Tables 3 and 4). The mean values obtained for samples analysed after low concentrations and after high concentrations showed no significant difference.

**Interferences by endogenic compounds**

Interference due to bilirubin was not observed in either method using concentrations of bilirubin up to 340 μmol/l.

Increasing concentrations of proteins retards the peroxidase activity. It has been reported that albumin acts as a competitive inhibitor [11]. With the Ektachem method, however, a slight increase in the glucose value is observed with increasing albumin concentration (Figure 8). Taking an albumin concentration of 80 g/l as the norm, at a concentration 120 g/l, a 6% higher, and at 20 g/l a 6% lower value for glucose is obtained. In routine diagnostics, however, this influence may be neglected. This is illustrated in Figure 9. The sera with protein concentrations above 100 g/l were taken from a patient suffering from paraproteinemia. The determination of urea in this sample was not affected by protein (Figures 8 and 10).

Curme et al [1] measured a glucose concentration in a haemolytic sample which was 7% lower than with a comparative method. The authors have not observed any interference up to concentrations of haemoglobin of 6.4 g/l. Also, no interference was observed with either of the two methods due to lipemic turbidity using triglyceridglycerol concentrations up to 32 mmol/l.

**Interferences by exogenic compounds**

Using the experimental procedure (Table 9) from a range of 46 compounds that were added to a pool of sera, significant interferences were only observed with the following compounds: chloramphenicol, tetracycline and ascorbic acid. Experiments were repeated adding these compounds to the same pool of sera in various, but lower concentrations which more closely approach those actually occurring in blood (Figures 11 and 12). This showed that only ascorbic acid produces a significant interference in the determination of glucose. The relevance of this interference cannot be

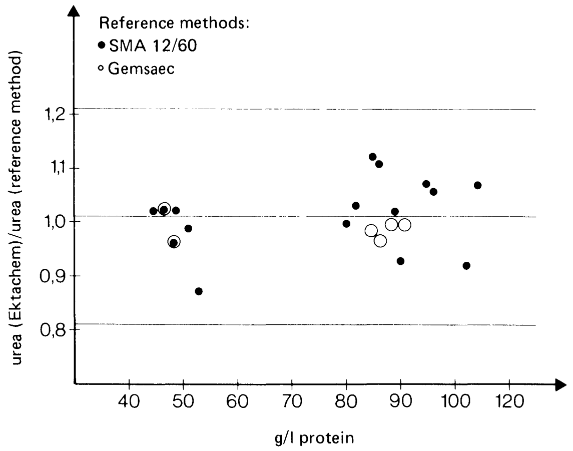

*Figure 10. Influence of protein on determination of urea concentration with the Ektachem system. The y-axis gives the values of the ratio between urea (Ektachem value in mmol/l) and urea (Gemsaec value in mmole/l). The range of confidence corresponds to the ($\bar{x}\pm 3s$) – range ($\bar{x}$ = mean value, s = standard deviation) of the 84 values that were used in Figure 5 and which had been obtained from visually transparent sera.*

assessed because the actual levels in patients' specimen is not actually determined in the author's laboratory. After intake of excessive amounts of ascorbic acid a plasma concentration of 47 mg/l (0.27 mmol/l) was measured [12]. A patient who was treated with 250 mg/kg methotrexate [13] provided no samples with a clear difference between the Ektachem and comparative methods (Table 10).

In accordance with a proposal by the FDA (Food and Drug Administration: Proposed Product Class Standard for Quantitative Measurement of Glucose in Serum or Plasma) various interfering compounds added to the sample sera must not alter the result by more than 5 mg/dl. Curme et al [1] maintain that out of 25 compounds added (most of which were not tested in this study) this requirement was not

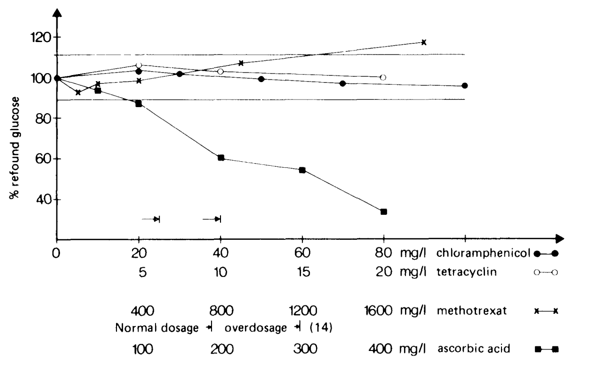

*Figure 11. Recovery values in percents of glucose in serum to which various compounds had been added. The range of confidence indicated at 88% and 112% corresponds to the (x̄ + 3s) – range (x̄ = mean value, s = standard deviation) that was obtained from 10 measurements of the same pool of serum without the added compounds.*

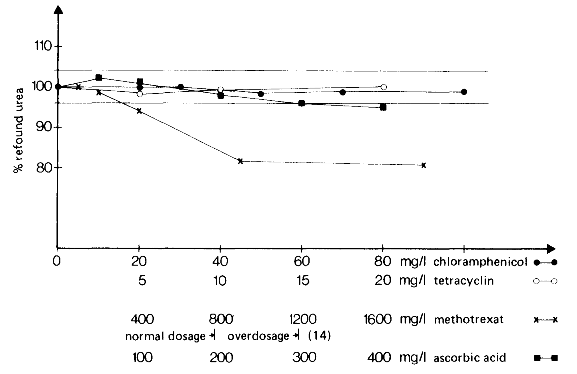

*Figure 12. Recovery values in percents of urea in serum to which various compounds had been added. For range of confidence see legend to Figure 11.*

**Table 10 Glucose and urea concentrations of a patient treated with infusions of methotrexat**

| methotrexat in serum μ mmol/l | glucose mmol/l | glucose mmol/l | urea mmol/l | urea mmol/l |
|---|---|---|---|---|
| 3,1 | 2,9 | 2,9 | 2,7 | 2,8 |
| 5,6 | 2,8 | 2,8 | 4,6 | 4,5 |
| 6,6 | 4,3 | 4,1 | 6,9 | 6,8 |
| 5,1 | 3,6 | 3,7 | 7,3 | 7,2 |
| 0,5 | 5,8 | 5,4 | 4,9 | 4,8 |
| 291,6 | 3,5 | 3,6 | 2,5 | 2,6 |
| 609,1 | 5,6 | 5,4 | 2,8 | 2,8 |
| 1095,1 | 6,5 | 6,5 | 3,2 | 3,1 |
| 123,1 | 6,6 | 6,7 | 3,7 | 3,8 |
| 275,4 | 3,8 | 3,9 | 2,2 | 2,1 |
| 6,0 | 3,3 | 3,4 | 2,1 | 2,0 |

**Table 11 Comparison of mean value and coefficient of variation (VK) for daily (A) and for one constant calibration (B) during 21 days (n=21) with the Ektachem system**

| method | glucose mean value | | | | urea mean value | | | |
|---|---|---|---|---|---|---|---|---|
| | A | B | $VK_A$ | $VK_B$ | A | B | $VK_A$ | $VK_B$ |
| Low Control | 3,37 | 3,37 | 2,97 | 2,67 | 3,28 | 3,35 | 3,05 | 1,79 |
| Wellcomtrol II | 11,65 | 11,97 | 2,58 | 2,59 | 29,28 | 29.77 | 2,42 | 1,75 |
| High Control | 28,35 | 28,19 | 1,94 | 1,21 | 37,66 | 37,66 | 2,04 | 1,91 |

fulfilled for ascorbic acid (100 mg/dl), thymol (100 g/l), and lipids (45 g/l).

Following a recommendation by the FDA (i.e. measurements lie within the 95% range of confidence as determined for the same pool of sera that has not been adulterated with pharmaceuticals), the manufacturer did not find a significant interference for urea for the following compounds: bilirubin (2+ mg/dl, 340 µmol/l), creatinin (10 mg/dl, 88 µmol/l), dextran 40 (1/gl), sodium salicylate (35 mg/l), uric acid (10 mg/l, 600 µmol/l).

Fluoride is an inhibitor for urease and the manufacturer recommends that any plasma samples with fluoride present should not be used. With both glucose and urea methods, a positive bias was observed for serum which has been spiked with sodium fluoride (Table 7).

### Practicability

The system is easy to operate, and it is almost impossible to make operating mistakes. The technical reliability of the instrument has been evaluated because an evaluation instrument was used, which was designed for glucose and urea determination only. The commercial instrument will be different. The control sera were analyzed and calibrations were carried out daily. The instrument settings remained fixed during the time of the experiments. The coefficients of variation were somewhat lower on average for a fixed calibration (Table 11). Therefore, one calibration per batch seems to be sufficient to obtain the necessary precision of results.

### Conclusion

The determination of concentrations of glucose and urea in human sera with the Ektachem system for analysis proved to be very precise and accurate. The range of linearity is sufficient. For routine medical diagnosis interferences so far observed can be tolerated. The poor agreement with the assigned values for controls also causes some concern and should be investigated further.

REFERENCES

[1] Curme, H.G., Columbus, R.L., Dappen, G.M., Eder, Th.W., Fellows, W.D., Figueras, J., Glover, C.P., Goffe, C.A., Hill, D.E., Lawton, W.H., Muka, E.J., Pinney, J.E., Rand, R.N., Sandford, K.J. and Wu, T.W. (1978), *Clinical Chemistry* **24**, 1335-1342.

[2] Sprayd, R.W., Bruschl, B., Burdick, B.A., Dappen, G.M., Elkenbery, J.N., Esders, Th.W., Figueras, J., Goodhue, Ch.T., La Rossa, D.D., Nelson, R.W., Rand, R.N., and Wu, T.W. (1978), *Clinical Chemistry* **24**, 1343-1350.

[3] Trinder, P. (1969), *Journal of Clinical Pathology* **22**, 158-164.

[4] Haeckel, R. und Mathias, D. (1974), *J. Clin. Chem. Clin. Biochem* **12**, 515-520.

[5] Haeckel, R. und Haeckel, H. (1972), *J.Clin. Chem. Clin. Biochem* **10**, 453-461.

[6] Haeckel, R., Sonntag, O., Külpmann, W.R. and Feldmann, U. (1979), *J.Clin. Chem. Clin. Biochem.*, **17.**

[7] Haeckel, R. (1976), *J.Clin. Chem. Clin. Biochem.* **14**, 101-107.

[8] National Committee for Clinical Laboratory Standards (1979) Protocol for establishing performance claims for clinical chemical methods. Proposed standard PSEP-3, USA.

[9] Anderson, T.W. (1958), An introduction of multivariate statistical analysis. J. Wiley, New York.

[10] Feldmann, U., Schneider, B. und Haeckel, R. (1980), *J. Clin. Chem. Clin. Biochem.*, in preparation.

[11] Gallati, H. (1977), *J. Clin. Chem. Clin. Biochem.* **15**, 699-703.

[12] Schrauzer, G.N. and Rhead, W.J. (1973), *Interna. J. Vit. Nutr. Res.* **43**, 201-211.

[13] Oellerich, M., Englehardt, P., Schaadt, M., und Diehl, V. (1979), *J. Clin. Chem. Clin. Biochem.*, in press.

### Decision criteria for selecting analytical instruments

In 1978 the Expert Panel on Instrumentation of the International Federation of Clinical Chemistry received a request from its Associate Member for New Zealand, to help in some way with guidance on the choice of analytical instrumentation. It was pointed out that relatively isolated communities such as in New Zealand, often find it impossible to choose rationally from the large amount of literature which presents a bewildering array with little indication as to which instrument is most suitable for a particular application.

The Expert Panel enlisted the cooperation of the International Union of Pure and Applied Chemistry's Commission on Automation, and together they prepared a series of papers to be presented at various international congresses, hopefully covering many of the factors to be taken into account when choosing instrumentation for the laboratory. These papers will be printed in the next issue of the Journal of Automatic Chemistry.

### The Journal of Automatic Chemistry

**The following papers have been accepted for publication and will be published in future issues.**

A semiautomatic system for subsampling heterogenous foods

Automatic analysis: the laboratory manager's problems

Use of a simple 8-bit microprocessor as a flexible sequence controller for developing laboratory automation

Decision criteria for selecting analytical instruments

The determination of pKa and partition data: an automated approach

